# Crystal Structure of a Complex between Amino and Carboxy Terminal Fragments of mDia1: Insights into Autoinhibition of Diaphanous-Related Formins

**DOI:** 10.1371/journal.pone.0012992

**Published:** 2010-09-30

**Authors:** Azin Nezami, Florence Poy, Angela Toms, Wei Zheng, Michael J. Eck

**Affiliations:** 1 Department of Cancer Biology, Dana-Farber Cancer Institute, Boston, Massachusetts, United States of America; 2 Department of Biological Chemistry and Molecular Pharmacology, Harvard Medical School, Boston, Massachusetts, United States of America; University of Oulu, Germany

## Abstract

Formin proteins direct the nucleation and assembly of linear actin filaments in a variety of cellular processes using their conserved formin homology 2 (FH2) domain. Diaphanous-related formins (DRFs) are effectors of Rho-family GTPases, and in the absence of Rho activation they are maintained in an inactive state by intramolecular interactions between their regulatory N-terminal region and a C-terminal segment referred to as the DAD domain. Although structures are available for the isolated DAD segment in complex with the interacting region in the N-terminus, it remains unclear how this leads to inhibition of actin assembly by the FH2 domain. Here we describe the crystal structure of the N-terminal regulatory region of formin mDia1 in complex with a C-terminal fragment containing both the FH2 and DAD domains. In the crystal structure and in solution, these fragments form a tetrameric complex composed of two interlocking N+C dimers. Formation of the tetramer is likely a consequence of the particular N-terminal construct employed, as we show that a nearly full-length mDia1 protein is dimeric, as are other autoinhibited N+C complexes containing longer N-terminal fragments. The structure provides the first view of the intact C-terminus of a DRF, revealing the relationship of the DAD to the FH2 domain. Delineation of alternative dimeric N+C interactions within the tetramer provides two general models for autoinhibition in intact formins. In both models, engagement of the DAD by the N-terminus is incompatible with actin filament formation on the FH2, and in one model the actin binding surfaces of the FH2 domain are directly blocked by the N-terminus.

## Introduction

Formin family proteins direct actin assembly in an array of cellular functions including cytokinesis, cell migration, and the establishment and maintenance of cell polarity[1]–[3]. Formins are present in all eukaryotes and many species express multiple isoforms of the protein[4]. They have in common the presence of the ∼400 residue formin homology 2 (FH2) domain, which directly mediates actin assembly[5]–[7]. Biochemically, the FH2 domain potently nucleates new, unbranched actin filaments and remains attached to the growing barbed end of the filament as additional actin subunits are incorporated. This activity is referred to as processive capping. The adjacent formin homology 1 (FH1) domain is a proline-rich segment that can accelerate actin assembly on the FH2 domain by recruitment of profilin-bound actin[8]–[10].

**Figure 1:**
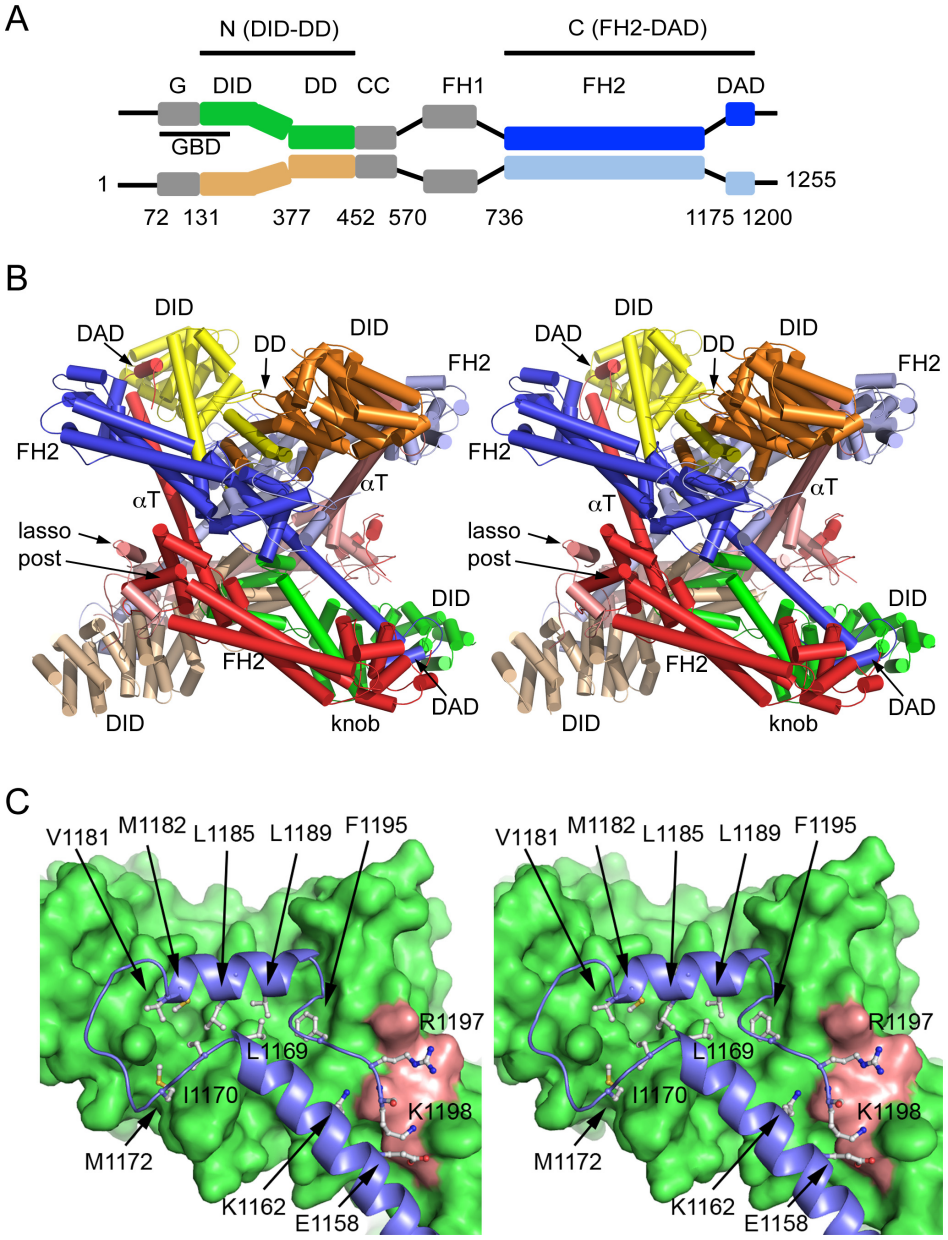
Structure of an autoinhibited mDia1 N+C complex. **A**, Dimeric domain structure of diaphanous-related formins. Dimerization is mediated by the DD, CC and FH2 domains as indicated. Numbering corresponds to murine mDia1; black bars indicate the extent of the crystallized N- and C-terminal fragments. Domains are colored as in panel B; this assignment of domains in the dimer corresponds to the proposed “trans” model of inhibition (see text, and Fig. 6A). Abbreviations: GBD, GTPase binding domain; G, GTPase binding subdomain; DID, DAD interacting domain; DD, dimerization domain; CC, coiled-coil domain; FH1, formin homology-1 domain; FH2, formin homology-2 domain; DAD, diaphanous autoregulatory domain. **B**, Stereo diagram showing the crystal structure of the tetrameric N+C mDia1 complex. Selected domains and subdomains are labeled. The bridge elements comprising the putative FH2 dimers are colored blue and light blue (“upper” FH2 dimer), or red and pink (lower FH2 dimer). The subunits of the N-terminal dimers are colored yellow and orange (upper dimer) or green and tan (lower dimer). Note that the N-terminal DID domains engage the DAD domains extending from helix αT of the more distal FH2 domain. See Figures 4 and S3 for additional representations of the tetramer. **C**, Stereo diagram depicting the interaction of the DID and DAD regions. The DID domain is shown in a surface representation and the interacting region of the C-terminus, including the end of αT and the DAD domain, is shown in a ribbon representation (blue, with selected sidechains shown in white). The core amphipathic DAD helix makes extensive hydrophobic contact with the DID domain as previously observed (residues Val 1181- Phe 1195). Note that the end of αT and the connecting loop also contribute hydrophobic interactions to the interface (Leu 1169, Ile 1170, and Met 1172). The basic portion of the DAD extends in an acidic groove between the DID and αT. The sidechains of Arg 1197 and Lys 1198 in this region approach, but do not make clearly defined salt-bridge interactions with acidic residues on the DID domain. The surface corresponding to acidic residues Glu 358, Asp361, Glu 362 and Asp 366 is shaded salmon.

Diaphanous-related formins (DRFs) are a subfamily of formins that are effectors of Rho-family GTPases[11]–[14]. The mammalian DRF Dia1 is important for stress fiber assembly and cell migration. Mice deficient in mDia1 exhibit myeloproliferative defects[15], [16] and impaired lymphocyte trafficking[17]. In the absence of interaction with GTP-bound Rho, mDia1 and other DRFs are maintained in an autoinhibited state by intramolecular interactions between their C-terminal diaphanous autoregulatory domain (DAD) and the interacting region in the N-terminus, termed the DAD interacting domain (DID). DRFs share a number of other functional and structural domains. The N-terminal GTPase binding domain (GBD) is formed from the helical G domain and a portion of the adjacent DID domain. These elements are followed by the dimerization domain (DD), a coiled-coil region, the FH1 and FH2 domains, and finally the C-terminal DAD domain (Figure 1A).

Extensive study of formins has revealed structures of the FH2 domain[18]–[21] and its mode of binding with actin[22], as well as structures of the regulatory N-terminal domains alone[23] and in complexes with GTP-bound Rho[24]or cdc42[25]. Biochemical studies and comparison of the Rho-bound structure with those of the N-terminal domains determined in complex with the DAD segment explain how the autoinhibitory DID/DAD interaction can be released by competition with GTP-bound Rho[26], [27]. Despite this relative wealth of biochemical and structural information, it remains unclear how the DID/DAD interaction inhibits actin assembly by the FH2 domain.

In order to better understand the mechanism of autoinhibition in mDia1 and other DRFs, we have reconstituted complexes containing the N-terminal regulatory region of mDia1 bound to a C-terminal fragment containing its FH2 and DAD domains for structural analysis. The FH1 domain was omitted from the C-terminal constructs because it is expected to be unstructured and therefore to impede crystallization. We crystallized and determined the structure of an “N+C” complex containing the N-terminal DID and DD domains (DID-DD) bound to the C-terminal FH2-DAD construct. In the crystal structure and in solution, these fragments form a tetrameric complex with the stoichiometry (DID-DD)_4_/(FH2-DAD)_4_. This tetrameric structure can be described as an interlocked complex of two N+C dimers. Formation of the tetramer is likely a consequence of the particular N-terminal construct employed, as we show that a nearly full-length mDia1 protein is dimeric, as are other autoinhibited N+C complexes containing longer N-terminal fragments that include a portion of the adjacent coiled-coil domain. The structure provides the first view of the intact C-terminus of a DRF, revealing the relationship of the DAD to the FH2 domain. Delineation of alternative dimeric N+C interactions within the tetramer provides two general models for autoinhibition in intact formins. In the “*cis*” model the N-terminal DID-DD region directly occludes the actin binding surface of the FH2 dimer while in the “*trans*” model the DID-DD region is maintained at a distance from the surface of the FH2 domain by the long αT helix, which extends from the FH2 domain to present the DAD segment for binding. In both the models, engagement of the DAD by the N-terminus is incompatible with actin filament formation on the FH2 domain.

## Results

### Preparation and analysis of mDia1 proteins and N+C complexes

We prepared amino-terminal fragments of murine Dia1 (mDia1) and reconstituted them into complexes with a carboxy-terminal fragment spanning the FH2 and DAD regions (residues 736–1200) of mDia1 for crystallization. The N-terminal fragments included the DID and DD domains, which are expected to be required for full autoinhibition[28], with and without the adjacent G domain and a portion of the coiled-coil region (Figure 1A, Table 1). All formed complexes with the C-terminal fragment that were stable on gel-filtration and/or ion exchange chromatography, and representative N-terminal preparations potently inhibited the activity of FH2-DAD in pyrene actin assembly assays (Figure S1). We refer to these as “N+C complexes” as they contain N- and C-terminal fragments of mDia1.

**Table 1:** SEC-MALS analysis of mDia1 proteins and complexes.

mDia1 Proteins and Complexes	Mw Determined by MALS (kDa)	Sequence Predicted Mw for monomer (kDa)		
		N+C	N	C
N: DID-DD, residues 131–458C: FH2-DAD, residues 736–1200	345	92	38	54
N: GBD-DID-DD, residues 72–458C: FH2-DAD, residues 736–1200	354	98	44	54
N: DID-DD-CC, residues 131–477C: FH2-DAD, residues 736–1200	195	94	40	54
C: FH2-DAD, residues 736–1200	109	-	-	54
mDia1-DID-C: residues 131–1255	278	125	-	-

**Figure 2:**
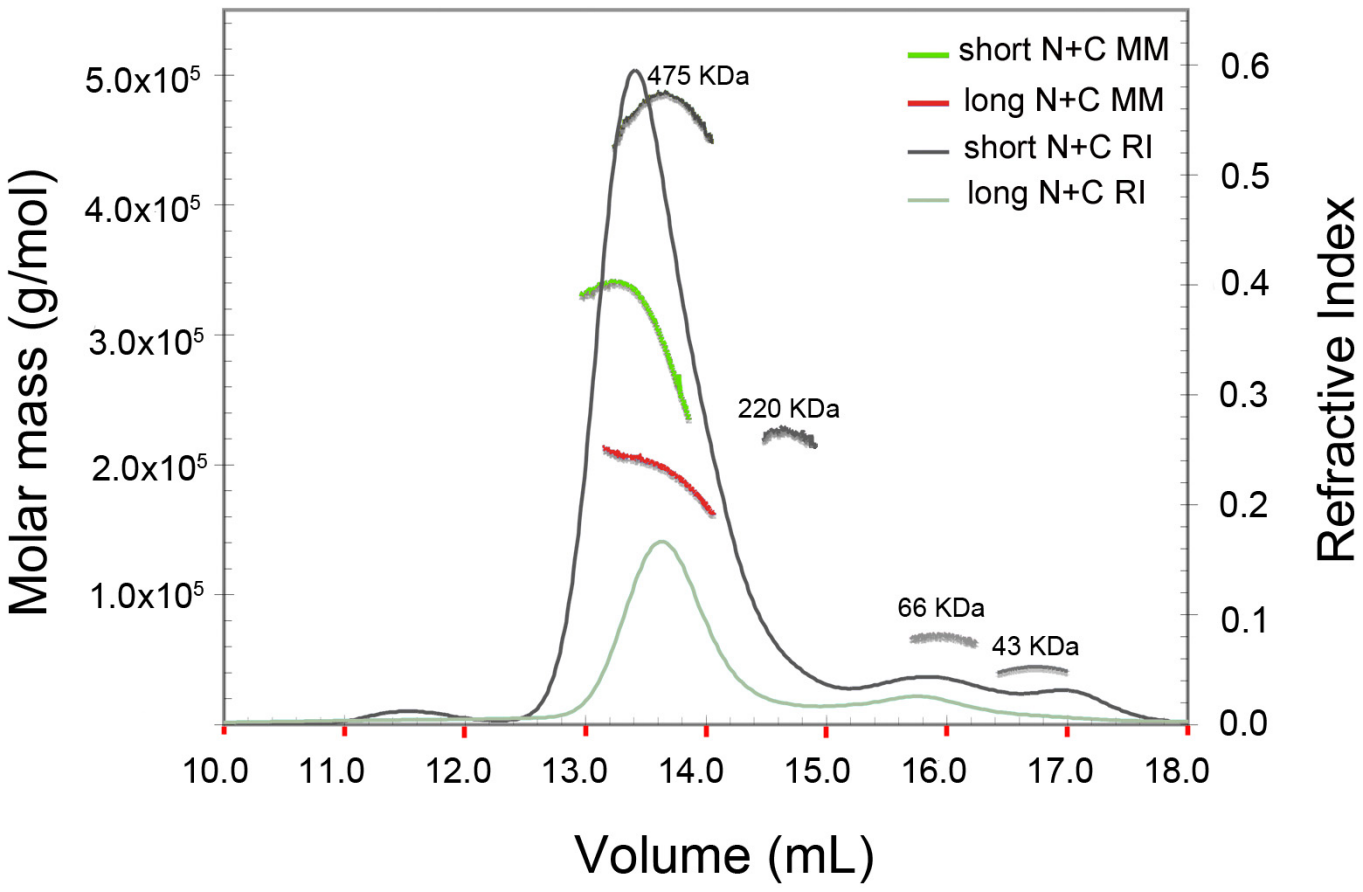
SEC-MALS analysis of mDia1 N+C complexes. Molar mass and refractive index are plotted versus elution volume from a Superose 6 size-exclusion column for N+C complexes containing residues 131–458 with 736–1200 of mDia1 (short N+C, green trace) or residues 131–477 with 736–1200 of mDia1 (long N+C, red trace). Derived molar masses are presented in Table 1. Molecular weight standards are overlaid in dark gray and correspond to ovalbumin (43 kDa), BSA (66 kDa), BAM (220 kDa), and Apo (475 kDa). Analysis was performed in the Biophysics Resource of the Keck Center at Yale University.

We analyzed selected N+C complexes using size-exclusion chromatography and multi-angle light scattering (SEC-MALS) to determine their approximate molar mass and thus their oligomeric state (Table 1). The N- and C- fragments are both known to be dimeric[23], [24], [29], and in the simplest model would be expected to form a dimeric complex, i.e. one N-terminal dimer is presumed to bind one C-terminal dimer via a dual DID/DAD interaction. This was indeed the case for the N+C complex containing the N-terminal fragment DID-DD-CC (residues 131–477). For this complex, MALS analysis yielded a molar mass of 195 kDa, consistent with a complex with the subunit composition (DID-DD-CC)_2_/(FH2-DAD)_2_. However, for both the G-DID-DD and DID-DD complexes, we observed molar masses most consistent with formation of tetrameric complexes, i.e. (DID-DD)_4_/(FH2-DAD)_4_. Despite their differences in subunit stoichiometry, the dimeric and tetrameric species eluted from the size-exclusion column (Superose 6) at similar volumes (Figure 2), suggesting that the dimer has an effective hydrodynamic radius similar to that of the tetramer. The marked drop in the apparent molar mass of the tetrameric species across its elution peak is suggestive of partial dissociation of the complex or the presence of a small quantity of a lower molecular weight complex (Figure 2, green trace). Analysis of these and other N+C complexes by polyacrylamide gel electrophoresis under non-denaturing conditions (NATIVE-PAGE) revealed that freshly prepared complexes contained a faster migrating, presumably dimeric species that partitioned with time into the more slowly migrating tetrameric band (Figure S2). In contrast, a construct that contained portions of the coiled-coil domain (G-DID-DD-CC, residues 63–518) exhibited only the faster migrating, presumably dimeric band (Figure S2).

**Figure 3:**
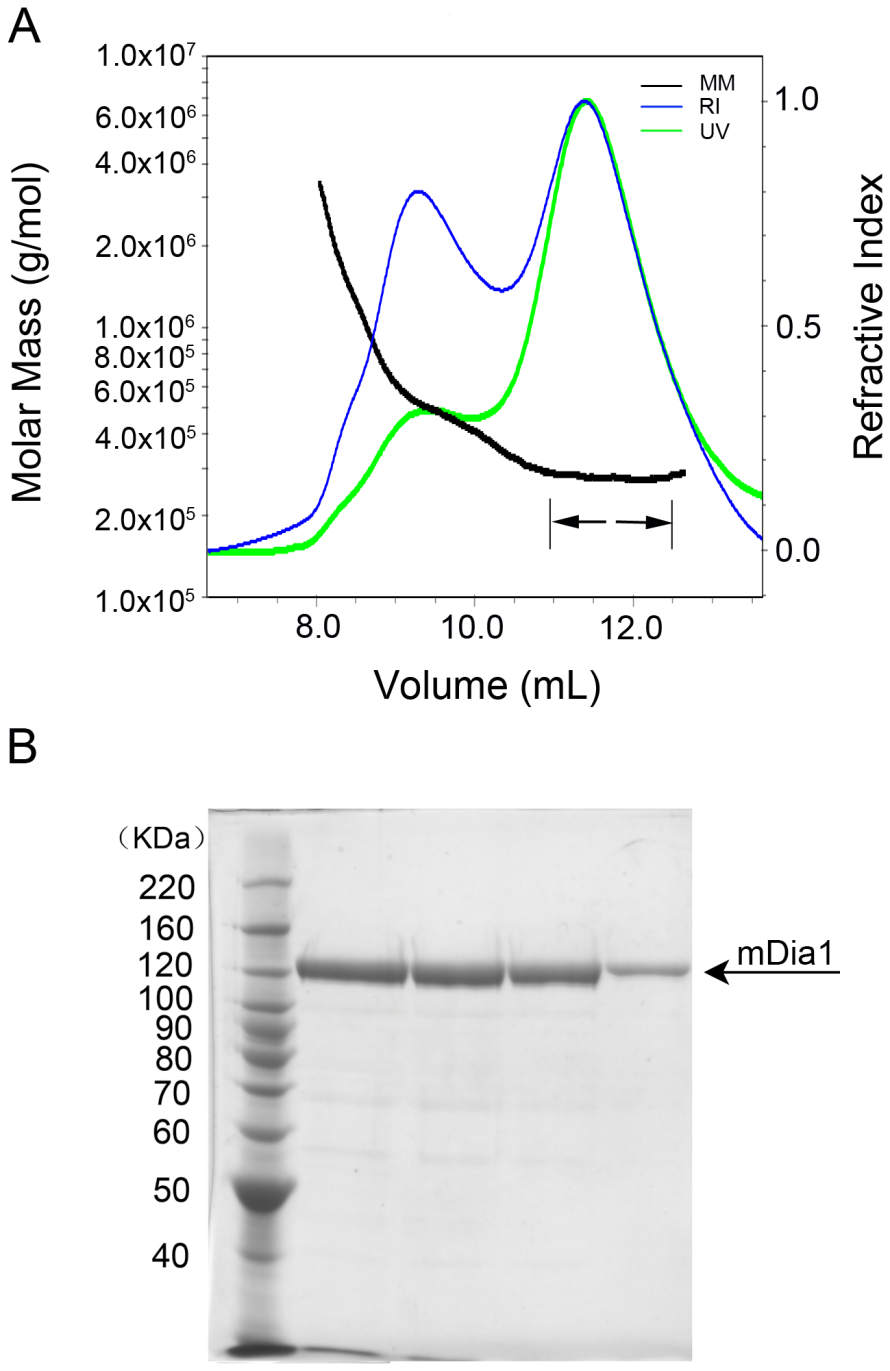
Purification and size analysis of mDia1-DID-C. Residues 131–1255 of murine mDia1 were expressed using an insect cell/baculovirus system, and purified by affinity and size-exclusion chromatography (see Materials and Methods). **A**, SEC-MALS analysis of the purified protein; molar mass (black trace, MM), refractive index (blue trace, RI) and absorbance at 280 nM (green trace, UV) are plotted versus elution volume from a Superdex 200 size-exclusion column. Calculated molar mass is presented in Table 1, and was measured across the volume indicated by the arrows. The earlier eluting peak contains a small amount of polydisperse, high molecular weight aggregates of mDia 131-1255 that were not completely removed in the prior size-exclusion chromatography step. **B**, Coomassie-stained SDS-PAGE analysis of Superdex 200 elution fractions of the purified mDia1-DID-C protein.

We also expressed and purified a nearly full-length fragment of mDia1 (mDia1-DID-C, residues 131–1255) for crystallization trials. In this construct, we omitted the non-conserved N-terminal region of the protein as well as the G domain, which is expected to be disordered in the autoinhibited state[23]. The mDia1-DID-C protein is dimeric, as judged by SEC-MALS analysis (Figure 3). Collectively, our data support the hypothesis that autoinhibited mDia1 is dimeric, and that truncation of the coiled-coil region in the reconstituted N+C complexes leads to formation of tetrameric species. We have been unable to crystallize the mDia1-DID-C protein or of any of the dimeric N+C complexes, but the tetrameric DID-DD/FH2-DAD complex crystallized readily, as described below.

### Crystal structure of an autoinhibited mDia1 complex

We obtained crystals of the DID-DD protein in complex with FH2-DAD in space group P2_1_ and determined the structure by molecular replacement using available structures of the mDia1 N-terminus and truncated FH2 domain as search models (see Materials and Methods). The structure was refined to a crystallographic R-value of 23.7% (R_free_ = 29.6%) using data extending to 3.2 Å resolution. Structure determination statistics are summarized in Table 2.

**Table 2:** Data collection and refinement statistics.*

**Data Collection**	
Space Group	P2_1_
Unit Cell Dimensions	a = 95.76 Å, b = 206.83 Å, c = 131.06 Å, β = 105.9°
Resolution (Å)	25–3.2 (3.3–3.2)
R_merge_ a	9.7 (57.5)
I/σI	14.26 (2.7)
Completeness (%)	99.5 (100)
Redundancy	3.8 (3.8)
**Refinement**	
Resolution (Å)	25–3.2
Number of Reflections	75584
R_work_/R_free_ b	23.7/29.6
Number of Protein Atoms	24185
Number of Water Molecules	48
Average B-factor	82.9
R.M.S.D. Bond Lengths (Å)	0.006
R.M.S.D. Bond Angles (°)	0.938
**Ramachandran Analysis**	
Most Favored (%)	95.6
Allowed (%)	99.9

*Highest resolution shell is shown in parentheses.

a Rmerge  =  Σ|Ii-<I>|/ΣIi, where Ii is the *i*th measurement of the intensity of an individual reflection or its symmetry-equivalent reflections and <I> is the average intensity of that reflection and its symmetry-equivalent reflections.

b Rwork  =  Σ||Fobs| − |Fcalc||/Σ|Fobs| for all reflections and *R*_free_  =  Σ||Fobs| − |Fcalc||/Σ|Fobs|, calculated on the 5% of data excluded from refinement.

The crystals contain two dimeric N+C complexes in the asymmetric unit, where each of these dimers is in turn composed of a dimeric N-terminal fragment bound to a dimeric C-terminal fragment via interactions of their respective DID and DAD domains. We refer to the crystallized structure as a tetramer, as it contains four copies of the N-terminal DID-DD chain and four copies of the FH2-DAD chain. As we discuss more fully below, we cannot unambiguously determine the pairing of C-terminal FH2 domain fragments into dimers. The tetramer has approximate 222 point symmetry, and is composed of two symmetrically interlocked N+C dimers, as illustrated in Figure 1B. Because its symmetry is non-crystallographic, the two-fold symmetry axes are not perfect as they are distorted by differing lattice contacts.

**Figure 4:**
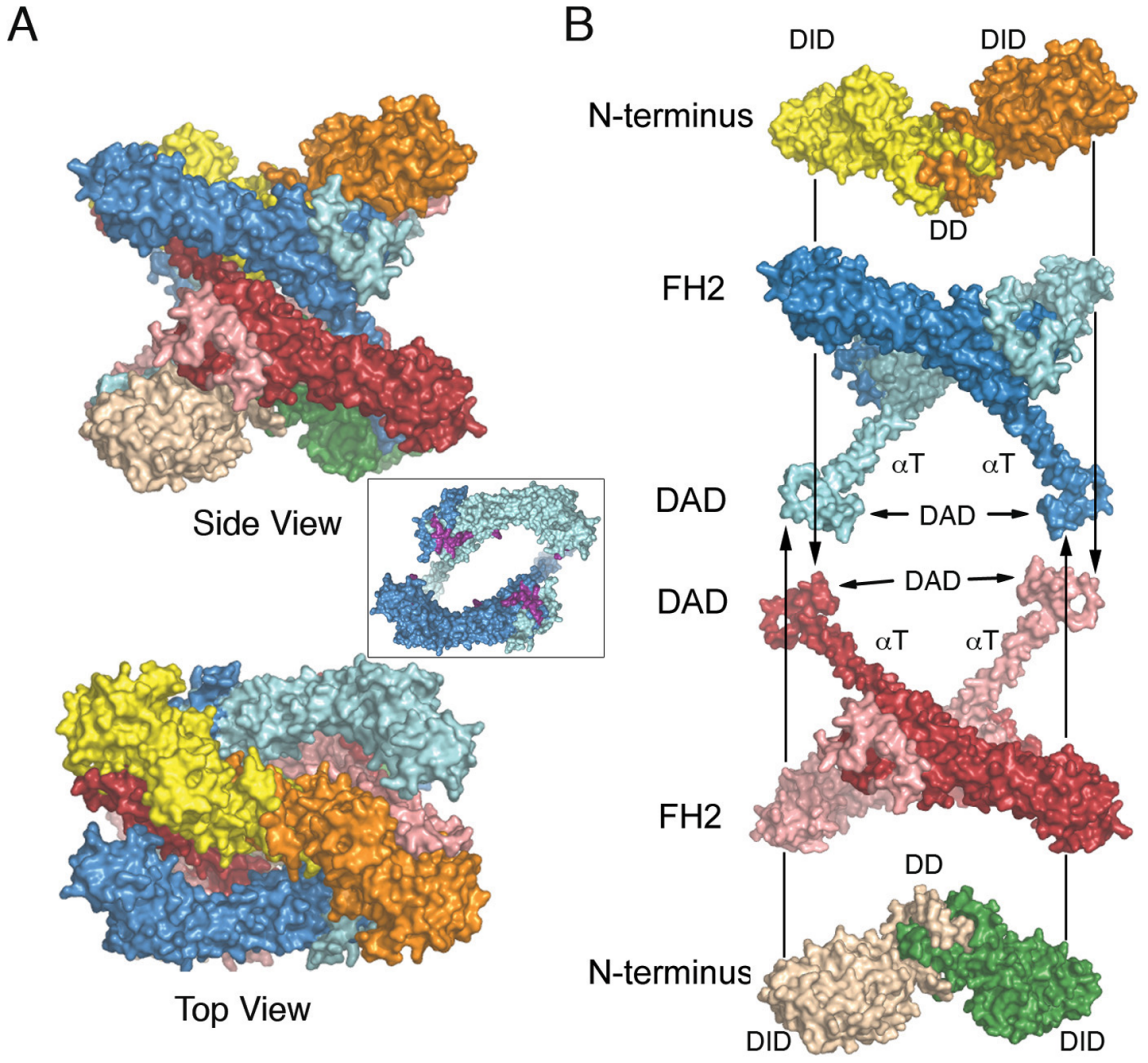
Surface views of the autoinhibited mDia1 N+C tetramer. **A**, Side and top views of the tetramer, colored as in Figure 1B. Inset: top view of one FH2 dimer, with the residues expected to bind actin shaded magenta. Note that packing of the N-terminal DID-DD dimers onto the FH2 domain blocks the actin-binding surface. **B**, “Exploded” view depicting construction of the tetramer.

The tetramer effectively consists of four layers; two FH2 dimers form the two central layers, while the N-terminal dimers pack on either side to form the two outer layers (Figure1B, Figure 4). This interlocked structure is held together primarily, if not entirely, by the interactions of the DID domains of each N-terminal dimer with the DAD domains emanating from the more *distal* FH2 dimer. Examination of the crystal lattice reveals no other plausible tetrameric assembly and we expect that the tetrameric structure we describe corresponds to that formed by these fragments in solution.

The FH2 domains exhibit the characteristic “tethered-dimer” architecture in which two rod-shaped domains are connected head-to-tail by flexible linker segments[18]. Topologically, the dimer forms a ring. Each side of the FH2 dimer (termed a hemidimer) is an elongated helical bundle containing the “knob” and “post” subdomains of one subunit and the “lasso” element of the adjoining subunit. The lasso of each subunit encircles the post of the other in a reciprocal manner to form the dimer. The lasso is followed in primary sequence by the flexible linker region, which leads in turn to the knob subdomain. An ∼85 Å long α-helix (αT) extends from the post of the FH2 domain, and the autoregulatory DAD domain segment is connected to the end of helix αT by a 10-residue loop segment.

In the present structure, all four flexible linkers are disordered, complicating assignment of the connectivity of the FH2 domain dimers. We describe and illustrate the nearest neighbor hemidimers as comprising the intact dimeric FH2 domains (the dark blue and light blue subunits as one dimer, the red and pink as the other as illustrated in Figs. 1 and 2). This assignment leaves a gap of ∼30 Å between the last ordered residue in the lasso (residue 806) and the first ordered residue of the knob of each chain (residue 828), a distance that would be easily spanned by the intervening 21 disordered residues. However, we note that an alternate assignment cannot be rigorously excluded (e.g. the dark blue and red subunits as a dimer). We consider the latter assignment unlikely, as the longer separation (56–60 Å in the four instances in the asymmetric unit) is at the limit that could reasonably be spanned by the intervening residues in a fully extended conformation. Furthermore, it would be virtually impossible for other DRFs with significantly shorter linkers to adopt such a conformation (e.g. four residues shorter in human Dia3).

The hemidimer contains two actin-binding surfaces – one on the knob subdomain and the other on the lasso/post. These two functional sites are proposed to bridge between actin subunits in the process of nucleation and processive capping. For this reason, the hemidimer is also referred to as a “bridge element” [22]. The knob actin-binding site is centered on Ile 845 in mDia1 (Ile 1431 in bni1) and the lasso/post site on Lys994 (Lys1601 in bni1) [18], [19], [22]. In the tetramer, the FH2 domains pack back-to-back, with their actin binding surfaces facing outward (up on one FH2 domain and down on the other in the orientation presented here). The long αT helices of each FH2 dimer extend diagonally between the bridge elements of the other FH2 dimer, thus presenting the DAD for binding to the contralateral DID domain.

As previously described [23], [24], the DID domain is composed of a series of armadillo repeats, a structural motif consisting of a repeating series of three helices arranged in a superhelical coil. The DID domain contains five armadillo repeats; the atypical fifth repeat leads to the dimerization domain via a long helix. The dimerization domain (DD) is formed by a zig-zag of three interdigitating helices from each subunit. The N-terminal DID-DD dimers in the present structure are approximately two-fold symmetric; the two halves of each DID-DD dimer are related by 178°–179° rotations. The isolated DID and DD domains superimpose well on the corresponding regions of the structure of the mDia1 N-terminus crystallized in complex with the isolated DAD peptide[26]. The DID domains of these structures superimpose with an RMSD of ∼0.65 Å, while the DD domains superimpose with an RMSD of ∼1.7 Å. Despite the fact that both structures are two-fold symmetric, the relative positions of their DID and DD domains differ greatly. If the present structure is superimposed on the mDia1 DAD complex (PDB ID 2BAP) based on the alignment of the DD domain, rotations of 43° and translations of approximately 15 Å are required to bring the DID domains into register. Considerable divergence in domain orientation has been noted in comparisons of previous structures of the DID-DD and G-DID-DD fragments of mDia1 [23], [24], [26].

The DAD is composed of a core helical region with the sequence motif “MDXLLXL” and an adjacent basic segment with the sequence “RRKR” in mDia1 [30]. The interactions of the DAD domain with the DID are essentially the same as previously described for structures containing the isolated DAD segment [26], [27], but additional interactions are observed for the basic C-terminal portion of the DAD. The core “MDXLLXL” motif in the DAD domain forms an amphipathic helix (residues 1180–1192) that packs into a conserved groove on the concave surface of the DID. Just beyond this helix, Phe 1195 packs into a hydrophobic cleft, as previously described[26], [27]. The basic segment extends along the DID domain, in a channel formed by the DID and helix αT (Fig 1C). This region has a highly acidic character, with four negatively charged residues on the surface of the DID and another contributed by αT. Electron density for the basic segment is weak, and somewhat divergent among the four copies in the tetramer. No interpretable density is present for the last two residues in the crystallized protein. Helix αT and the loop that connects it to the DAD also contribute to hydrophobic contacts with the DID and DAD regions (Fig 1C).

### Structural Comparison mDia1, Daam1 and Bni1 FH2 domains

**Figure 5:**
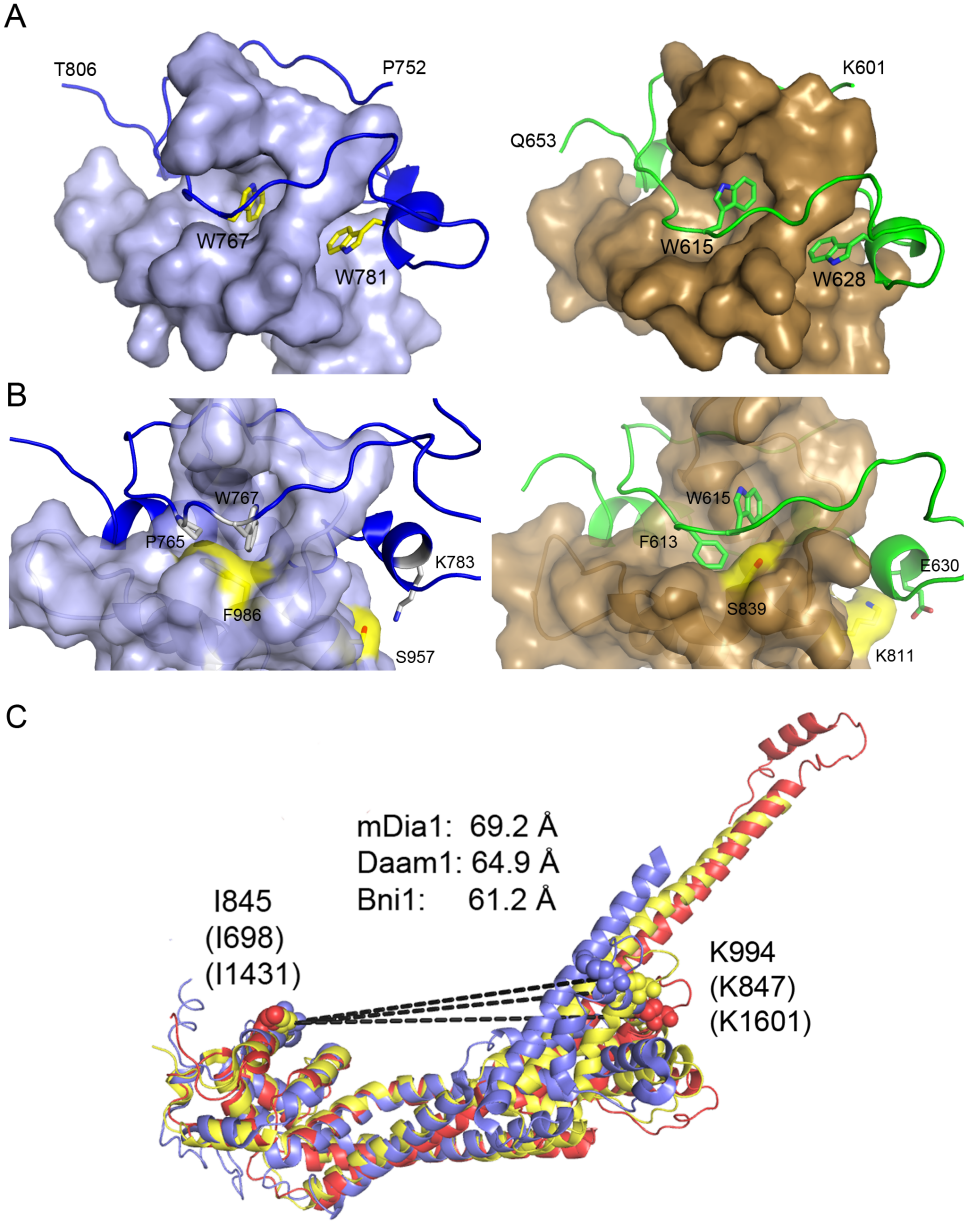
Comparison of FH2 domain structures. **A**, Lasso-post interface of mDia1 (left panel) and Daam1 (right panel). Both mDia1 and Daam1 exhibit the same general dimerization mode first described for yeast formin Bni1, in which the lasso region of one subunit encircles the post of the other. Conserved tryptophan residues in the lasso insert into pockets in the post. **B**, Despite the generally conserved mode of interaction, distinct differences in the lasso-post interface in mDia1 (left) as compared with Daam1 (right) are likely to preclude heterodimerization of their FH2 domains. For example, Pro765 in the mDia1 lasso replaces Phe613 in Daam1. This change is accompanied by substitution of Phe896 in mDia1 for Ser839 in Daam1 and a reorientation of the sidechain of Trp767. Additionally, Lys783 in the lasso hydrogen bonds with Ser957 in the post in mDia1, which corresponds to Lys811 in the post of Daam1. **C**, Superposition of the mDia1 (red), Daam1 (yellow) and Bni1 (blue) hemidimers reveals considerable divergence in the relative spacing of knob and post actin-binding sites. Structures are superimposed based on the knob subdomain. Although actin-binding residues in the knob and lasso/post regions independently superimpose well among these FH2 domains, their spacing differs by as much as 8 Å, as measured between key actin-binding residues in the knob (Ile 845 in mDia1) and post (Lys 994 in mDia1).

The structure of a monomeric fragment of the mDia1 FH2 domain has previously been described[19] and also compared with Bni1 and Daam1[20], [21]. However, the present structure provides the first reported view of the functional mDia1 FH2 domain with the lasso/post interface intact, affording additional insights into conserved and divergent features of the FH2 domain. As expected, the general mode of dimerization in which the lasso of one subunit loops around the post region of the other is conserved among mDia1, Daam1 and Bni1[18]. In particular, two highly conserved tryptophan residues in the lasso insert into pockets in the post domain as previously documented in Bni1 and Daam1 (Figure 5A). However, there are a number of sites of divergence in the interface. For example, Phe 986 in the post of the mDia1 FH2 domain replaces a much smaller serine in Daam1 (Figure 5B). This change in the post is accommodated by a compensatory change in the lasso – Pro765 in the mDia1 lasso replaces the phenylalanine residue found in the corresponding position in Daam1. Similarly Lys783 hydrogen bonds with Ser957 in the post in mDia1, but in Daam1 the corresponding residues are Glu630 in the lasso and Lys811 in the post (Figure 5B). These and other divergences in the lasso/post interface may have evolved in part to preclude heterodimerization of FH2 domains [31], [32].

Although the knob and post regions of mDia1, Daam1 and Bni1 independently superimpose well, there is considerable variation in the spacing between knob and post actin binding sites among these formins (as much as 8 Å, Figure 5C). This difference arises largely from differences in the orientation of the knob subdomain with respect to the rest of the bridge element[20]. This divergence is unlikely to result entirely from flexibility in the bridge element; this measurement differs by less than 1 Å among crystallographically independent copies of the FH2 hemidimer in the present structure. Thus this variation in spacing suggests that precise positioning of actin subunits by knob and post sites within the bridge element is not critical for the mechanism of nucleation or processive capping.

## Discussion

**Figure 6:**
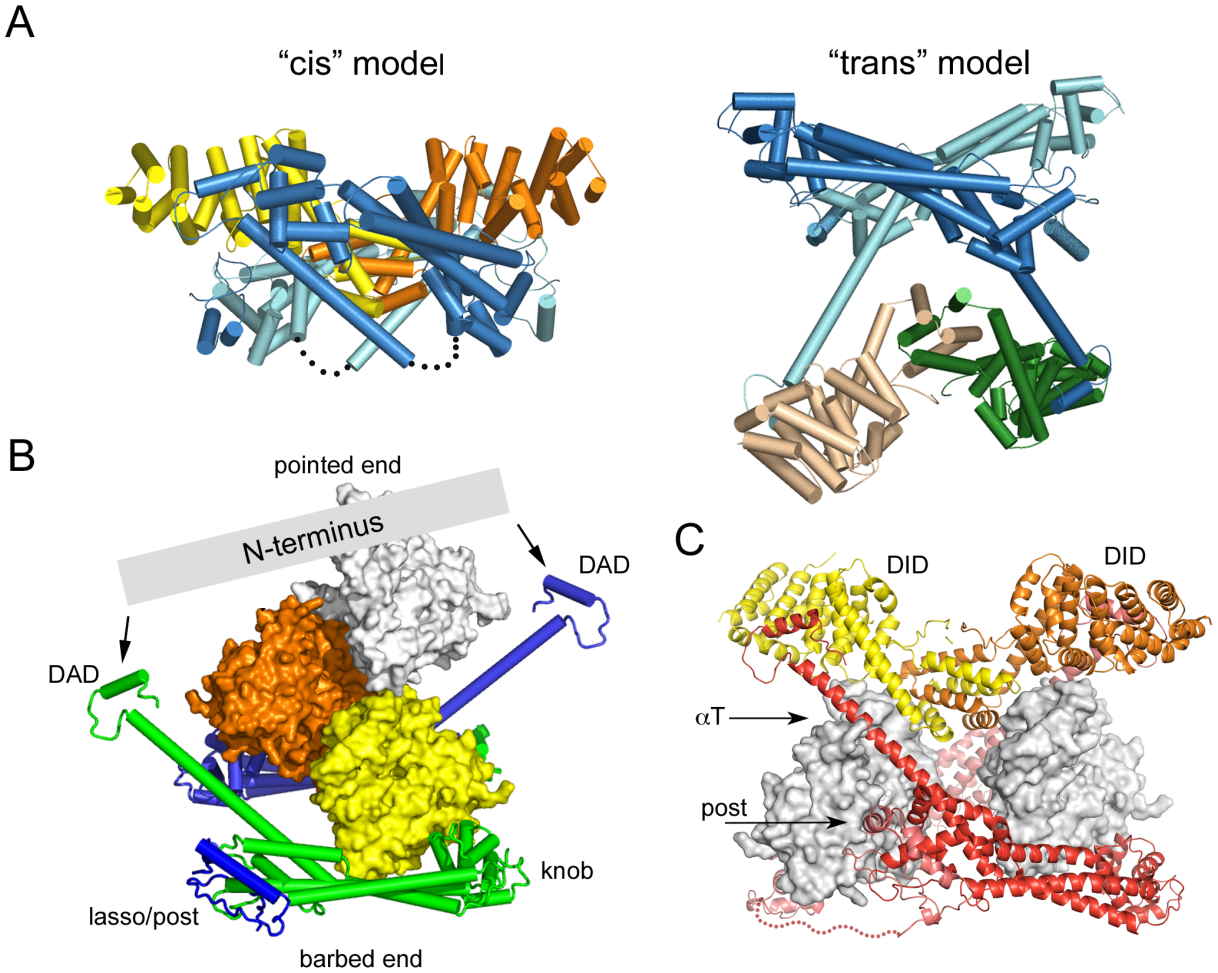
Speculations on mechanisms of autoinhibition in intact diaphanous-related formins. **A**, The autoinhibited tetramer structure suggests two general models for an autoinhibited dimer. Structures are colored as in Figure 1B. In the *cis* model (left), the N-terminus packs into the FH2 domain, as in the top half of the tetramer. This arrangement would require the αT Helix of each hemidimer to fold back (as indicated by the dotted lines) in order to allow binding of the DAD segment to the adjacent N-terminus. In this model, formation of the tetramer represents a “domain swap” in which the DAD and C-terminal portions of αT exchanged positions with the corresponding portions of the juxtaposed C-terminus to create the interlocked tetramer. The *trans* model maintains the DID/DAD interaction found in the tetramer; it is simply the complex of the C-terminal fragment with the N-terminus that engages its DAD region (e.g., the upper FH2 domain with the lower N-terminus). **B**, The structure of the FH2+DAD region of mDia1 is docked on actin as in the structure of the Bni1/actin complex. Simultaneous engagement of the DAD segments by the dimeric N-terminus in almost any conceivable conformation or orientation (represented by the gray bar) would be expected to block addition of more than one or two actin subunits, and therefore to lead to inhibition of productive nucleation and processive capping. **C**, Actin subunits are docked on the knob subdomain of each bridge element of an FH2 dimer, with the N-terminus engaged as in the *trans* model. Actin subunits were docked on mDia1 by superposition of its knob subdomain with that of Bni1 in the Bni1/actin structure [22]. The superposition and docking reveals minor steric clashes between actin and the αT and post regions of the adjacent bridge element, but these could likely be relieved by flexibility in the FH2 linkers (shown as dotted lines), the loops that lead into the DAD domains, and in the N-terminus itself. However, note the large gap between actin subunits. Distortion of the FH2 dimer by engagement of the N-terminus is likely to interfere with stabilization of filament-like interactions between actin subunits, which are thought to be required for effective nucleation.

The autoinhibited tetramer structure suggests two general models for autoinhibition in an intact, dimeric DRF (Figure 6A). These are essentially the two ways in which an N+C dimer can be dissected from the tetrameric structure, given our qualified assignment of nearest-neighbor hemidimers as a connected FH2 domain dimer. The *cis* model consists of the upper half of the tetramer, with the N-terminus packed against the adjacent FH2 domain. This model would require the αT Helix of each hemidimer to break and/or fold back (as indicated by the dotted lines) in order to allow binding of the DAD segment to the adjacent N-terminus. If this model is correct, formation of the tetramer represents a “domain swap” in which the DAD and C-terminal portions of αT exchanged positions with the corresponding portions of the juxtaposed C-terminus to create the interlocked tetramer. In the *cis* model the mechanism of autoinhibition is clear; the actin-binding surfaces of the bridge elements are directly occluded by the N-terminal domains (see Figure 4A).

The *trans* model is the complex of the C-terminal fragment with the N-terminus that engages its DAD region (e.g., the upper FH2 domain with the lower N-terminus, Figure 5A). One can also consider a more general version of the *trans* model, in which the N- and C-terminal fragments are not constrained to adopt precisely the orientation found in the tetramer. This is particularly relevant to the N-terminus; as discussed above, relative orientations of the DID domains varies greatly between available crystal structures and in the presence of the coiled-coil region they have been observed to adopt a highly asymmetric configuration[23]. (*Nota Bene:* In both *cis* and *trans* models, the interaction is intramolecular in the context of the intact formin dimer. C*is* and *trans* to refer to the action and position of the N-terminus relative to the FH2 domain.)

How is actin assembly inhibited in the generalized *trans* model? Although the actin binding surfaces of the bridge element are not directly blocked in this model, the *trans* interaction could lead to autoinhibition by at least two mechanisms. First, spanning of the two DAD domains by the N-terminus, in essentially any plausible conformation or orientation would block addition of more than one or two actin subunits in a nascent filament or nucleus (Figure 6B). Second, productive nucleation is thought to require templating of two or three actin subunits by the FH2 domain in a filament-like orientation[22]. Distortion and/or rigidification of the FH2 dimer by the bound N-terminus are likely to preclude productive engagement of actin. Docking of actin on the knob of each bridge element in the dimer leaves the actin subunits separated by several Ångstroms, lacking the actin-actin contacts thought to be critical for nucleation (Figure 6C). Finally, we note that in both the *cis* and *trans* models, sequestration of the DAD segment by binding with the DID domain may also contribute directly to autoinhibition, as the DAD region also appears to play a positive role in actin assembly (B. Goode, personal communication).

Because the same DID/DAD interactions stabilize the autoinhibited state in both the *cis* and *trans* models, it is not straightforward to design a mutagenesis strategy to discriminate between them. A more definitive understanding of the mechanism of autoinhibition will require structural investigations of an intact, dimeric autoinhibited formin.

## Materials and Methods

### Protein expression and purification

The DNA sequence encoding residues 131–458 (N-terminus) and 736–1200 (C-terminus) of mDia1 was amplified by PCR and ligated into a modified pET vector containing a His_6_ tag with a TEV cleavage site. Expression of the fusion protein in *E. coli* BL21 (DE3) was induced by IPTG and was allowed to proceed overnight at 23°C. The cells were harvested and resuspended in 50 mM Tris (pH 8), 150 mM NaCl, and 200 µg/ml lysozyme and were stored at −80°C. Upon thawing and after the addition of 1 mM PMSF and 2 mM TCEP, the cells were lysed by sonication and cleared by centrifugation. The proteins were purified separately by metal-affinity chromatography on a Ni-chelating column. To form the N+C complex, excess C-terminal protein was added to N-terminal protein and the affinity tag was removed by overnight incubation at 4°C with TEV protease. The N+C complex was purified by anion exchange chromatography (HiTrapQ) and buffer exchanged into 20 mM Tris (pH 8), 100 mM NaCl and 2 mM TCEP (PD10 column), and concentrated to 5–7 mg/ml. Analogous procedures were followed to prepare complexes containing alternate N-terminal fragments listed in Table 1.

For insect cell expression and purification of mDia1-DID-C, a PCR fragment encoding residues 131–1255 of murine mDia1 was subcloned into a modified pTriEx transfer vector (Novagen) using 5′-BglII and 3′-XholI restriction sites. The modified transfer vector (pTriExGST-mDia1-DID-C) drives expression of the mDia1 protein as a GST-fusion with an intervening TEV protease cleavage site. The pTriExGST-mDia1-DID-C plasmid was co-transfected into Sf9 cells with linearized baculoviral DNA (BacVector-3000, Novagen). The primary virus was harvested after one week and was subsequently amplified, plaque purified and used to prepare aliquots of Baculovirus-Infected Insect Cells (BIICs)[33]. For protein production, five 800 mL shake flasks of Sf9 cells were infected using BIICs aliquots when cells reached a density of 2.0*10^6^ cells/ml. Cells were harvested by centrifugation 72 h after infection, and were lysed with a detergent-containing lysis buffer (20 mM Tris pH 8.0, 150 mM NaCl, 5% glycerol, 2 mM TCEP and 1% NP40). The lysate was clarified via centrifugation at 40,000 *g* for 1 h, and the resulting supernatant was incubated for 3 h with glutathione-Sepharose beads. The bead-bound fusion protein was cleaved by overnight incubation with TEV protease. TEV was removed by adsorption to an ion exchange column (MonoS, GE Healthcare) and the mDia1 protein was further purified by a size-exclusion chromatography (Superdex200, GE Healthcare) in storage buffer (20 mM Tris pH 8.0, 150 mM NaCl, and 2 mM TCEP).

### Light scattering analysis

Size-exclusion chromatography Multi-angle Light Scattering (SEC-MALS) experiments for mDia1 N+C and mDia1 C-terminal only constructs were carried out at HHMI & W.M. Keck Foundation Biotechnology Resource Laboratory, Yale University. The N+C complexes were prepared for SEC-MALS as described above for crystallization; 1∶1 complexes were isolated by anion exchange chromatography (HiTrapQ) prior to SEC-MALS analysis. Protein samples (1 mg/ml, 0.3 ml) were injected onto a Superose 6 size exclusion column at a flow rate of 0.3 ml/min in 20 mM HEPES pH 7.4, 150 mM NaCl, 1 mM EDTA and 2 mM DTT. Data were evaluated using the Zimm model for static light scattering data fitting. All proteins are monodisperse and their experimental molecular weights and calculated molecular weights for monomer are shown in Table 1.

The mDia1-DID-C protein sample (0.1 mg/ml, 0.3 ml) was injected onto a Superdex 200 size exclusion column attached to a GE AKTA purifier at a flow rate of 0.5 ml/min in 20 mM Tris pH 8.0, 150 mM NaCl, 2 mM TCEP. The eluted peak was analyzed using a Wyatt miniDAWN TREOS multi-angle light scattering instrument and a Wyatt Optilab rEX differential refractometer. Data were evaluated in ASTRA 5.3.4 software using the Zimm model as above.

### Crystallization and Structure Determination

The N+C complex was crystallized at 20°C by hanging-drop vapor diffusion against 0.2 M sodium malonate (pH 7.0) and 10–14% PEG 4000 as precipitant. Crystals grew overnight and were cryo-protected with 20% glycerol in drop mother liquor prior to flash freezing in liquid nitrogen. Diffraction data were collected at beamline X29 at the NSLS (Table 2) and processed with XDS [34]. Crystals belonged to the monoclinic space group P2_1_ and contained four N-terminal DID-DD chains and four C-terminal FH2-DAD chains in the asymmetric unit. Phases were determined by molecular replacement using the program Phaser[35], [36] using residues 133-371 of chain A of the mDia1 DID/DAD complex (PDB:2F31) and residues 829–1150 of chain A of the mDia1 FH2 domain (PDB: 1V9D) as the search models. The correct solution for the DID model yielded a log(likelihood) gain of 1092 in the 25–3.5 Å resolution range. With the N-terminal model in place a correct solution for three of the four FH2 domains yielded a log(likelihood) gain of 1550. The final FH2 domain could then be placed into NCS averaged 2F_o_-F_c_ maps. The programs Coot [37] and O [38] were used to build the model into the 2F_o_-F_c_ and F_o_-F_c_ maps in iterative rounds of NCS refinement with Refmac5 [39] and CNS v1.2 [40]. The final model contains residues 134–451 of the N terminal portion of mDia1, residues 745–1198 of the C terminal portion of mDia1, and 48 water molecules. Among the four copies of each polypeptide in the asymmetric unit, there is a variable break in the chain in the region of residues 192–200 in the N-terminal fragment and between residues 806–828 in the C-terminal fragment. The structure has been refined to an R value of 23.7% (R_free_  = 29.6%) with good stereochemistry using data extending to 3.2 Å resolution.

## Supporting Information

Figure S1 Pyrene actin assembly assays with N+C complexes. A, The mDia1 FH2-DAD protein (residues 736–1200, blue traces) potently nucleates actin assembly as previously described[41], but the tetrameric N+C complex (residues 72–458 plus 736–1200, green traces) shows little activity above that of actin alone (black trace). B, Comparison of actin assembly activity of various N-terminal constructs in complex with FH2-DAD. Note that the 72-458 and 63-462 complexes form tetramers, while the 63-518 complex is dimeric (see Figure S2). N+C complexes were pre-formed and purified (see Materials and Methods), and actin filament assembly assays were performed using 1% pyrene-labeled rabbit skeletal muscle actin as described [42]. Briefly, 2 µM G-actin was mixed with F-buffer (10 mM Tris, pH 7.5, 0.7 mM ATP, 0.2 mM CaCl2, 2 mM MgCl2, 50 mM KCl, 0.2 mM DTT) alone or with the indicated mDia proteins. Pyrene fluorescence was monitored using an excitation wavelength of 365 nm and an emission wavelength of 407 nm in a fluorescence spectrophotometer.
(0.37 MB TIF)

Figure S2 Native-Page analysis of N+C mDia1 complexes. The indicated complexes of N- and C-terminal fragments of mDia1 were analyzed by polyacrylamide gel electrophoresis under non-denaturing conditions using a 4–15% gradient gel on a PhastSystem (Pharmacia). Complexes in lanes 1,2,5 and 6 were co-purified as described (see Materials and Methods), while those in lanes 3 and 4 were prepared by combining separately purified N- and C-terminal fragments immediately prior to analysis on Day 0 (left panel). The same preparations were re-examined after five days (right panel). Freshly prepared complexes containing N-terminal residues 63-462 or 72-458 contained a faster migrating, presumably dimeric species that partitioned with time into the more slowly migrating tetrameric band (lanes 1 and 4, compare Day 0 vs. Day 5). In contrast, a construct that contained portions of the coiled-coil domain (residues 63–518) exhibited only the faster migrating, presumably dimeric band (lane 2, Day 0 vs. Day 5). Aliquots of the 72-458/736-1200 and 131-458/736-1211 complexes that were used for crystallization (and were stored at 5 mg/ml) were mostly tetrameric (lanes 5, 6).
(1.18 MB TIF)

Figure S3 Additional views of the tetrameric mDia1 complex. A, Stereodiagram of the tetramer, colored as in Figure 1B. The terminal residues of one N-terminal (DID-DD) and one C-terminal (FH2-DAD) fragment are labeled to facilitate following the path of the polypeptide chain. B, Subunits labeled in A are shown in isolation (and in the same orientation). These N- and C-terminal fragments could plausibly correspond to those of a continuous polypeptide chain in the trans model of autoinhibition (see text). Note the break in the chain between residues 811 and 828 in the C-terminal fragment; this corresponds to the flexible linker in the FH2 domain that is disordered in the present structure. C, Stereodiagrams of the tetramer in a ribbon representation. The view in the lower panel is rotated by 90° about the vertical axis.
(5.16 MB TIF)
