# Supplementary figures and images for: Crystal Structure of a Complex between Amino and Carboxy Terminal Fragments of mDia1: Insights into Autoinhibition of Diaphanous-Related Formins
Source: PLoS One. 2010 Sep 30;5(9):e12992. doi: 10.1371/journal.pone.0012992 (PMC2948013; doi:10.1371/journal.pone.0012992)

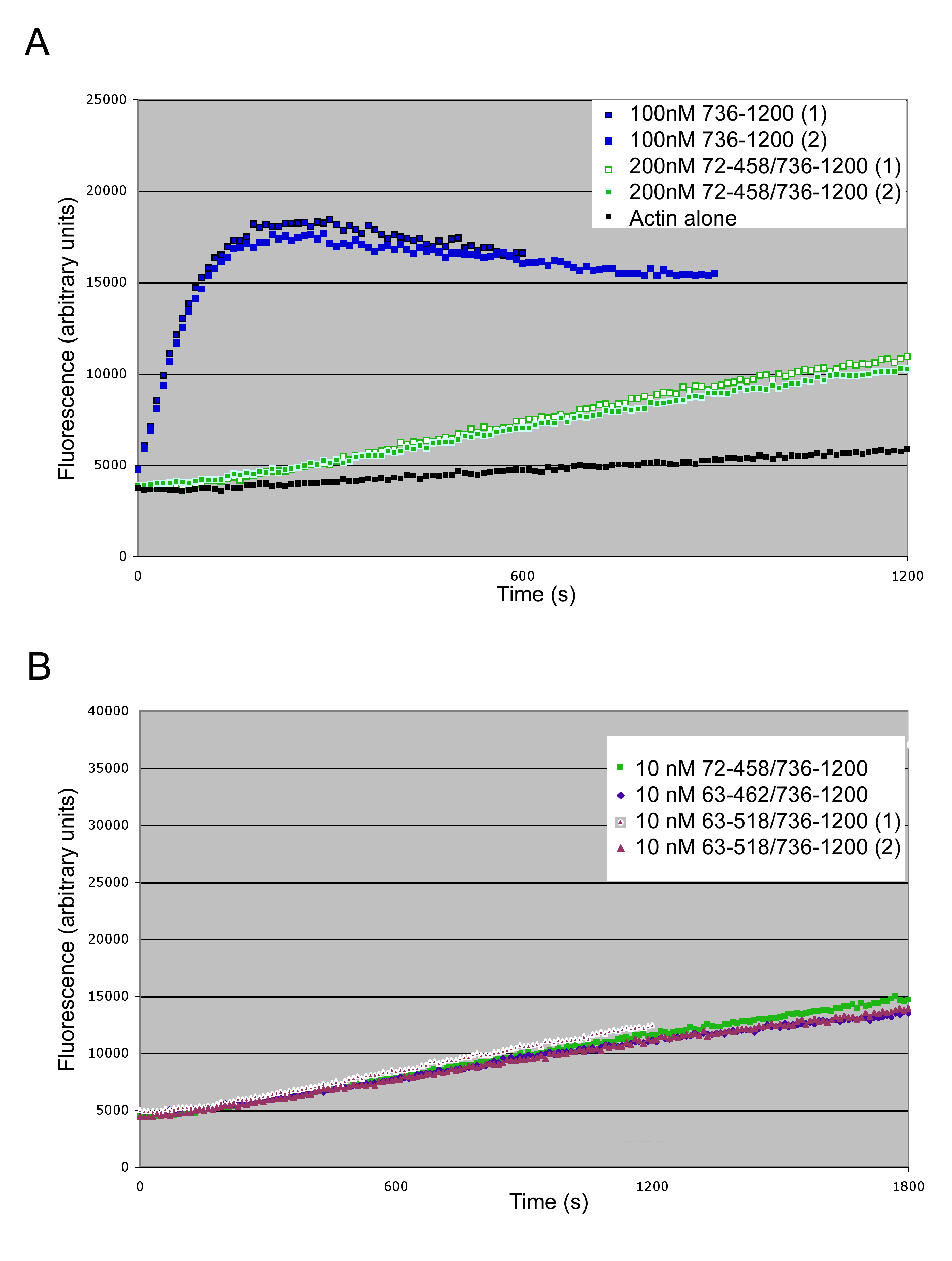

Supplement: Figure S1 — Pyrene actin assembly assays with N+C complexes. A, The mDia1 FH2-DAD protein (residues 736–1200, blue traces) potently nucleates actin assembly as previously described[41], but the tetrameric N+C complex (residues 72–458 plus 736–1200, green traces) shows little activity above that of actin alone (black trace). B, Comparison of actin assembly activity of various N-terminal constructs in complex with FH2-DAD. Note that the 72-458 and 63-462 complexes form tetramers, while the 63-518 complex is dimeric (see Figure S2). N+C complexes were pre-formed and purified (see Materials and Methods), and actin filament assembly assays were performed using 1% pyrene-labeled rabbit skeletal muscle actin as described [42]. Briefly, 2 µM G-actin was mixed with F-buffer (10 mM Tris, pH 7.5, 0.7 mM ATP, 0.2 mM CaCl2, 2 mM MgCl2, 50 mM KCl, 0.2 mM DTT) alone or with the indicated mDia proteins. Pyrene fluorescence was monitored using an excitation wavelength of 365 nm and an emission wavelength of 407 nm in a fluorescence spectrophotometer. (0.37 MB TIF) [file pone.0012992.s001.tif]

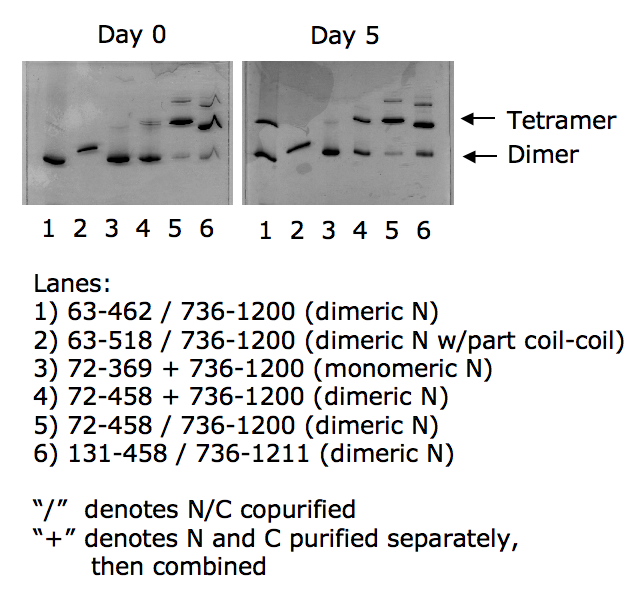

Supplement: Figure S2 — Native-Page analysis of N+C mDia1 complexes. The indicated complexes of N- and C-terminal fragments of mDia1 were analyzed by polyacrylamide gel electrophoresis under non-denaturing conditions using a 4–15% gradient gel on a PhastSystem (Pharmacia). Complexes in lanes 1,2,5 and 6 were co-purified as described (see Materials and Methods), while those in lanes 3 and 4 were prepared by combining separately purified N- and C-terminal fragments immediately prior to analysis on Day 0 (left panel). The same preparations were re-examined after five days (right panel). Freshly prepared complexes containing N-terminal residues 63-462 or 72-458 contained a faster migrating, presumably dimeric species that partitioned with time into the more slowly migrating tetrameric band (lanes 1 and 4, compare Day 0 vs. Day 5). In contrast, a construct that contained portions of the coiled-coil domain (residues 63–518) exhibited only the faster migrating, presumably dimeric band (lane 2, Day 0 vs. Day 5). Aliquots of the 72-458/736-1200 and 131-458/736-1211 complexes that were used for crystallization (and were stored at 5 mg/ml) were mostly tetrameric (lanes 5, 6). (1.18 MB TIF) [file pone.0012992.s002.tif]

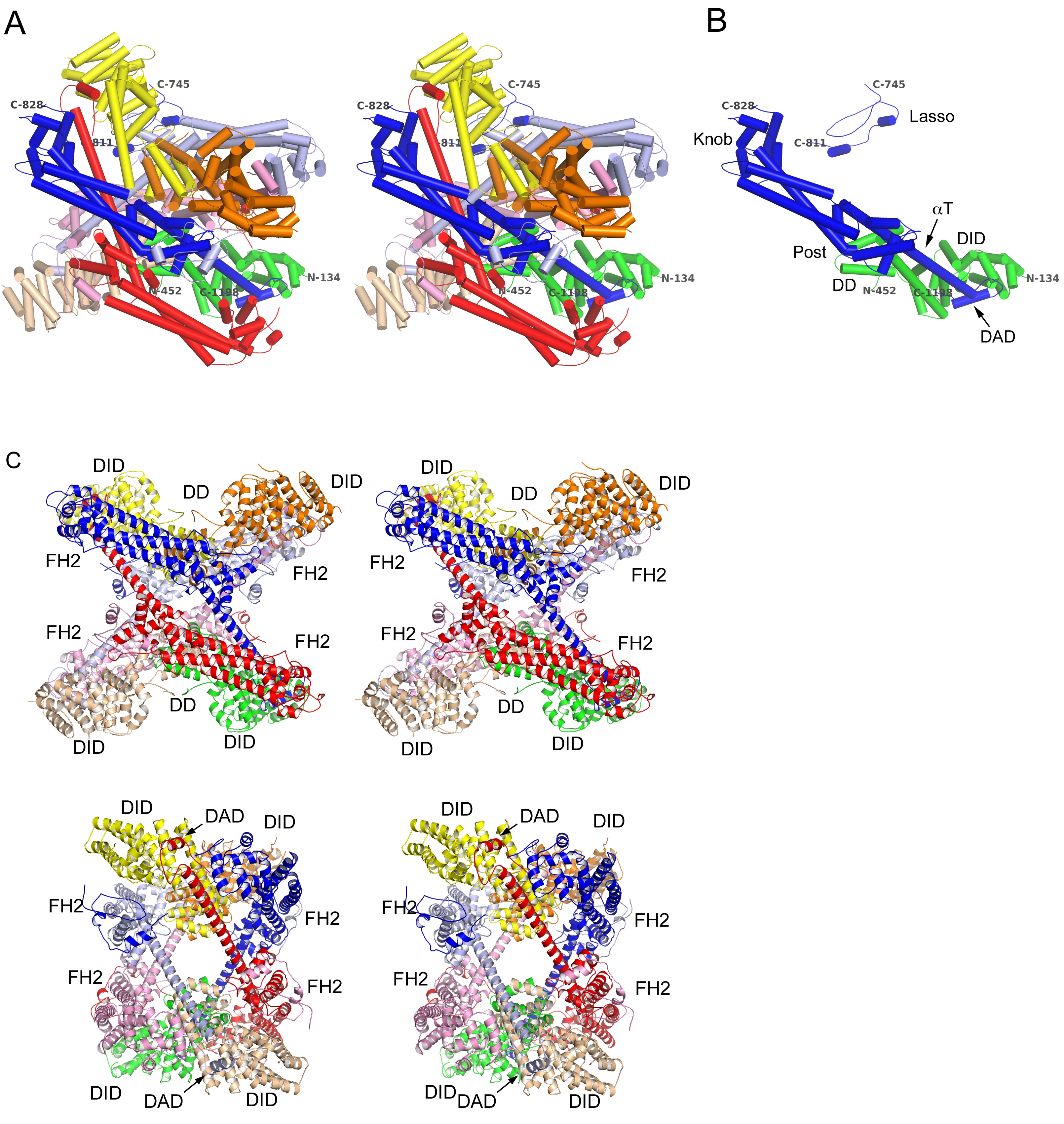

Supplement: Figure S3 — Additional views of the tetrameric mDia1 complex. A, Stereodiagram of the tetramer, colored as in Figure 1B. The terminal residues of one N-terminal (DID-DD) and one C-terminal (FH2-DAD) fragment are labeled to facilitate following the path of the polypeptide chain. B, Subunits labeled in A are shown in isolation (and in the same orientation). These N- and C-terminal fragments could plausibly correspond to those of a continuous polypeptide chain in the trans model of autoinhibition (see text). Note the break in the chain between residues 811 and 828 in the C-terminal fragment; this corresponds to the flexible linker in the FH2 domain that is disordered in the present structure. C, Stereodiagrams of the tetramer in a ribbon representation. The view in the lower panel is rotated by 90° about the vertical axis. (5.16 MB TIF) [file pone.0012992.s003.tif]
